# 

**Published:** 1889-04

**Authors:** 


VOL. X. APRIL, 1889. No. 4.

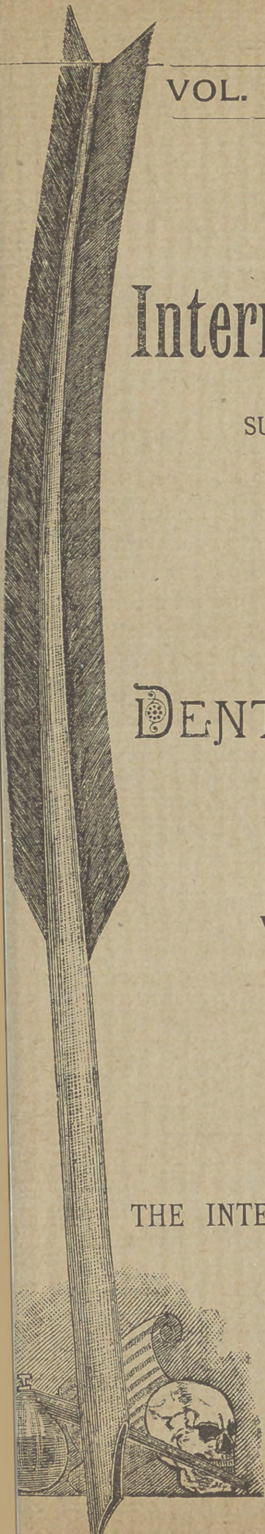

THE

### International Dental Journal:

SUCCESSOR TO THE INDEPENDENT PRACTITIONER.

A MONTHLY PERIODICAL

DEVOTED TO

### DENTAL AND ORAL SCIENCE.

W. XAVIER SUDDUTH, M. D., D. D. S.,
EDITOR.

PUBLISHED BY

THE INTERNATIONAL DENTAL PUBLICATION COMPANY,

**51 WEST 37th STREET, NEW YORK CITY,**

—AND—

**1215 FILBERT STREET, PHILADELPHIA.**

*Entered at Philadelphia Post Office as second-class reading matter.*

$2.50 Per Annum in Advance, Single Copies, 25 Cents.

